# A Microfluidic Platform for Evaluating Neutrophil Chemotaxis Induced by Sputum from COPD Patients

**DOI:** 10.1371/journal.pone.0126523

**Published:** 2015-05-11

**Authors:** Jiandong Wu, Craig Hillier, Paul Komenda, Ricardo Lobato de Faria, David Levin, Michael Zhang, Francis Lin

**Affiliations:** 1 Department of Physics and Astronomy, University of Manitoba, Winnipeg, MB, R3T 2N2, Canada; 2 Department of Biosystems Engineering, University of Manitoba, Winnipeg, MB, R3T 2N2, Canada; 3 Department of Biological Sciences, University of Manitoba, Winnipeg, MB, R3T 2N2, Canada; 4 Department of Immunology, University of Manitoba, Winnipeg, MB, R3E 0T5, Canada; 5 Seven Oaks General Hospital, Winnipeg, MB, R2V 3M3, Canada; 6 Rizhao Hospital of Traditional Chinese Medicine, Rizhao, China; University of Leuven, Rega Institute, BELGIUM

## Abstract

Chronic Obstructive Pulmonary Disease (COPD) is a common lung disease characterized by breathing difficulty as a consequence of narrowed airways. Previous studies have shown that COPD is correlated with neutrophil infiltration into the airways through chemotactic migration. However, whether neutrophil chemotaxis can be used to characterize and diagnose COPD is not well established. In the present study, we developed a microfluidic platform for evaluating neutrophil chemotaxis to sputum samples from COPD patients. Our results show increased neutrophil chemotaxis to COPD sputum compared to control sputum from healthy individuals. The level of COPD sputum induced neutrophil chemotaxis was correlated with the patient’s spirometry data. The cell morphology of neutrophils in a COPD sputum gradient is similar to the morphology displayed by neutrophils exposed to an IL-8 gradient, but not a fMLP gradient. In competing gradients of COPD sputum and fMLP, neutrophils chemotaxis and cell morphology are dominated by fMLP.

## Introduction

Neutrophils are the ‘first responders’ to the site of inflammation and play important roles in the human innate immune system [1]. Chemotaxis, a process whereby cells migrate toward a chemical concentration gradient, critically mediates neutrophil recruitment in tissues [2]. The biological mechanisms of neutrophil chemotaxis are highly complex and involve sophisticated cellular machinery for gradient sensing and migration [3]. Neutrophils express multiple chemoattractant receptors and can respond to individual chemoattractant gradients or their combinations [4]. Terminal chemoattractants, such as N-formyl-methionyl-leucyl-phenylalanine (fMLP), and intermediate chemoattractants, such as interleukin 8 (IL-8), trigger hierarchical chemotactic signaling and define distinct migration characteristics in neutrophils [5]. IL-8 can induce bi-directional migration of neutrophils in a dose-dependent manner [6]. Furthermore, spatiotemporal variations of IL-8 gradient profiles result in different migratory responses of neutrophils [7]. Taken together, neutrophil chemotaxis is coordinated by complex environmental factors. Changes of these factors can modulate cell migration patterns and their associated physiological outcomes.

Indeed, disorders of neutrophil chemotaxis are associated with various diseases such as asthma, chronic obstructive pulmonary disease (COPD), sepsis, diabetes, and kidney failure [8–12]. Among them, COPD is one of the most common lung diseases resulting from narrowed airways that cause breathing difficulty [13]. Long-term exposure of lungs to noxious particles or gases, such as cigarette smoke, is considered as the main cause for COPD [13]. In 2002, COPD was the fifth leading cause of death worldwide and the World Health Organization (WHO) has predicted that COPD will become the third leading cause of death by 2030 [14]. Spirometry, which measures the ratio of the forced expiratory volume in the first second to the forced vital capacity (FEV1/FVC), is the current "gold standard" for COPD diagnosis [15]. The main limitation of spirometry is that it requires patient cooperation, which can be difficult for patients with severe conditions, or if the patients cannot comprehend and follow the procedures [16]. Secretion of chemotactic factors such as IL-8 and leukotriene B4 (LTB_4_) in the airways recruit neutrophils, leading to inflammation and tissue damage [11, 17–20]. Neutrophil chemotaxis induced by sputum of COPD patients has been demonstrated *in vitro*, and is associated with COPD progression [21]. Measurement of neutrophils, or the level of chemotactic factors responsible for recruiting neutrophils, in the patient’s sputum have been proposed as new diagnostic markers for COPD [11]. Thus, it is of great interest to further assess neutrophil chemotaxis to the sputum of COPD patients as a means of diagnosing COPD at the cellular function level.

Previous neutrophil chemotaxis studies in COPD were based on traditional cell migration assays, such as the transwell assay and the under-agarose assay [20, 22]. Variations among these cell migration assays, and their common limitation in gradient control, has made it difficult to obtain reliable chemotaxis measurements, and in some cases has lead to contradictory results [21, 23]. Clinical factors such as the patient’s medical history and condition, methods of sample acquisition and processing, and the choice of control reference further complicate the interpretation of the chemotaxis results. Microfluidic devices provide a powerful new experimental tool for quantitative single cell migration and chemotaxis analysis owing to their ability to configure well-defined chemical concentration gradients, as well as advantages in miniaturization, low reagent consumption, real-time visualization of cell migration, and high throughput [16, 24–30]. Indeed, microfluidic devices have been widely used for studying neutrophil migration and chemotaxis [30]. In addition to research applications to better understand the biological mechanisms underlying neutrophil chemotaxis [3, 31], several studies have reported the use of microfluidic devices for testing neutrophil chemotaxis with clinical samples for different disease models such as burn injury and chronic inflammation [32, 33]. Most recently, a microfluidic system was successfully used to rapidly test neutrophil chemotaxis from a drop of blood for asthma diagnosis [34].

In the present study, we developed a new microfluidic platform for evaluating neutrophil chemotaxis to the sputum samples from COPD patients. This platform allowed us to quantitatively characterize COPD sputum induced neutrophil chemotaxis. Furthermore, we employed this system to assess the potential of neutrophil chemotaxis as a new clinical measure for COPD that may enable future point-of-care applications.

## Materials and Methods

### Sputum sample preparation

Ethics approval for obtaining sputum samples from COPD patients and healthy subjects (Protocol number: J2012:140) was granted by the Joint-Faculty Research Ethics Board at the University of Manitoba. Informed written consent form was obtained from all participants by the recruiting staff employed at the Seven Oaks General Hospital in Winnipeg following the procedures approved by the ethics board. The participants were given the opportunity to review the consent form in details and discuss with the recruiting staff for questions before they answer the questions in the consent form and provide their consenting signatures in the form. Spontaneous sputum samples were collected from COPD patients (based on spirometry and physician diagnosis) and healthy control subjects at the Seven Oaks General Hospital in Winnipeg, Manitoba, Canada. For this initial proof-of-concept study, sputum samples from a small cohort of total 5 COPD patients and 5 healthy control subjects were used. The clinical descriptors of the participants are summarized in Table 1. The sputum samples were transferred to 1.5 mL Eppendorf tubes and mixed with an equal volume of 0.1% dithiothreitol (Fisher Scientific). The samples were gently vortexed and placed in a water bath at 37°C for 15 minutes (min) to homogenize them. The samples were then centrifuged at 2800rpm for 10 min. The supernatants were collected and centrifuged again at 3000 rpm for 5min to completely remove cellular components. The supernatants were stored at -80°C before use. The supernatants were diluted by 10X in migration medium (RPMI-1640 with 0.4% BSA) for chemotaxis experiments.

**Table 1:** The clinical descriptors and the chemotaxis measurements of the COPD patients and healthy control subjects in this study.

	Age	Gender	FVC(L)	FEV1(L)	FEV1/FVC (×100%)	C.I. (SEM)	Speed (μm/s)(SEM)
**COPD patients**	74	F	1.68	0.59	35	0.533(0.028)	0.129(0.007)
	72	F	1.93	1.05	54	0.442(0.022)	0.156(0.007)
	57	F	1.89	0.73	39	0.317(0.04)	0.118(0.01)
	63	M	2.6	1.56	60	0.352(0.062)	0.252(0.029)
	67	F	1	0.75	75	0.304(0.039)	0.135(0.008)
**Healthycontrol subjects**	41	M	NA	NA	NA	0.298(0.02)	0.154(0.006)
	53	M	NA	NA	NA	0.144(0.013)	0.212(0.005)
	26	M	NA	NA	NA	0.265(0.045)	0.277(0.017)
	29	F	NA	NA	NA	0.191(0.045)	0.272(0.028)
	64	F	NA	NA	NA	0.194(0.031)	0.192(0.011)

### ELISA for measuring IL-8 concentration in sputum samples

The IL-8 concentration in the sputum samples was measured using a commercial ELISA kit (Cedarlanelabs, ON) and a multi-plate reader at 450 nm (Synergy 4 HT). Each sputum sample was assayed in duplicate.

### Neutrophil preparation

To reduce the variation of neutrophils among different donors, we used the neutrophils from one third-party healthy blood donor for each set of cell migration experiments. Ethics approval for obtaining blood samples from healthy human donors (Protocol number: J2009:030) was granted by the Joint-Faculty Research Ethics Board at the University of Manitoba. Informed written consent form was obtained from all participants by the recruiting staff employed at the Victoria General Hospital following the procedures approved by the ethics board. The participants were given the opportunity to review the consent form in details and discuss with the recruiting staff for questions before they answer the questions in the consent form and provide their consenting signatures in the form. Peripheral blood mononuclear cells (PBMCs) were removed using standard gradient centrifugation method. The remaining contents in the blood samples after PBMCs removal were mixed with Dextran-500, which partially separates the red blood cells (RBCs) to the bottom portion of the blood sample. The neutrophil enriched upper portion of the blood sample was transferred to a new tube and RBCs in the sample were further removed using RBC lysis buffer (BioLegend, CA). Isolated neutrophils were washed with RPMI-1640 for 2 times and re-suspended in RPMI-1640 medium before experiments. Neutrophils were used for experiments within 8 hours (hrs) after isolation.

### Microfluidic device preparation

The microfluidic device was fabricated in Polydimethylsiloxane (PDMS) (Dow Corning, MI) using the standard photolithography and soft-lithography methods. Briefly, the device pattern was designed in Freehand (Macromedia) and the design was printed to a transparency film as the photomask. The design was then patterned in SU-8 photoresist (MicroChem, MA) on a silicon wafer by UV exposure through the photomask. The photoresist pattern was used as a positive mold to make PDMS replicas. Holes (5mm diameter) were punched out of PDMS as solution wells and the outlet. The cell migration channel is 350 μm in width and 100 μm in height. The PDMS replica was then plasma bonded to a glass slide. The microfluidic device was coated with fibronectin (0.25 mg/mL, BD Biosciences, MA)(this high coating concentration was used to achieve the required surface coating density due to the low height of the microfluidic channel [28]) for 1 hr followed by 0.4% BSA blocking for another hour at room temperature.

### Chemotaxis experiment setup

**Fig 1:**
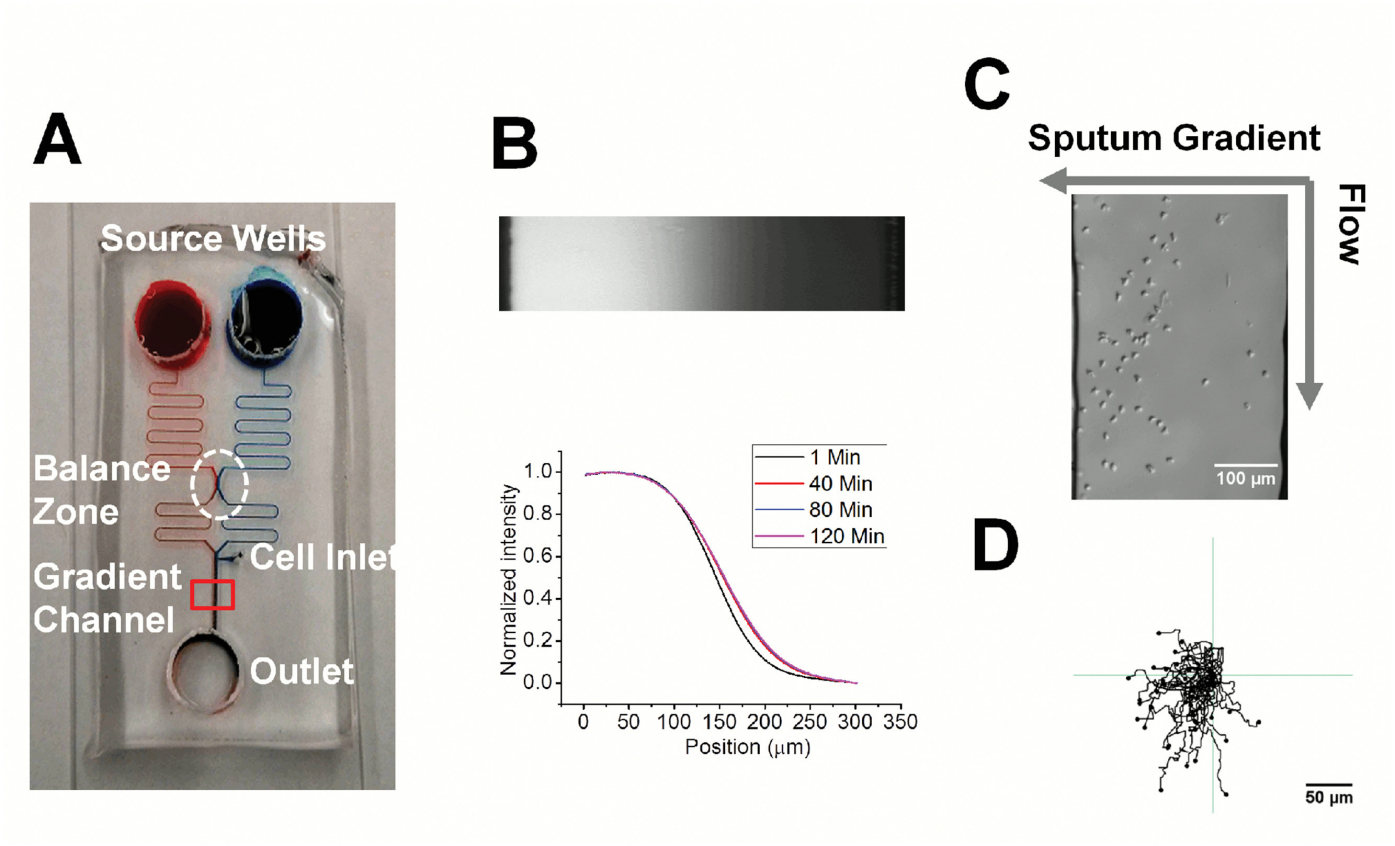
Illustration of the microfluidic device for testing neutrophil chemotaxis. **(A)** Illustration of the microfluidic gradient generator with a pressure balance zone. Red and blue dyes were injected into the device for visualization of the channels; **(B)** The fluorescent image of the FITC-Dextran gradient in the mirofluidic channel and the corresponding gradient profiles at different time points; **(C)** A representative image of neutrophils in a COPD sputum gradient in the microfluidic channel at the end of the 20min migration experiment; **(D)** The corresponding cell tracks for the experiment in **(C)**.

After seeding neutrophils onto the fibronectin-coated microfluidic channel, the two inlet wells of the device were filled with 100 μL of chemoattractant-containing migration medium and the migration medium alone (RPMI-1640 with 0.4% BSA) respectively. IL-8 (72 amino acid form, 208-IL-010, R&D Systems), fMLP (F3506, Sigma-Aldrich), and sputum supernatant were used as chemoattractants. Then the outlet well was emptied. The pressure difference between the inlet and outlet wells establishes flows of the solutions from the inlet wells into the channels. Mixing of the flows generates a gradient of chemoattractant in the microfluidic channel (Fig 1A and 1B). The gradient was calibrated by adding FITC-Dextran 10 kDa (final concentration of 5 μM, Sigma-Aldrich, MO) to one of the solutions and measuring the fluorescent profile. The device was placed under an inverted microscope (Nikon Ti-U) with an environmental control enclosure chamber (InVivo Scientific) to maintain the temperature of the microscope stage at 37°C. Differential interference contrast (DIC) time-lapse images of cell migration were captured every 10 seconds (s) for 15–20 min.

### Data analysis

#### Cell migration analysis

The neutrophil migratory behaviors in different conditions were analyzed by previously established manual tracking methods [24]. Specifically, the individual cell trajectories were obtained using the “Manual Tracking” plug-in in NIH ImageJ. The movement of cells was quantitatively evaluated by 1) the Chemotactic Index (C.I.), which is the ratio of cell displacement toward the gradient to the total migration distance; and 2) the average speed (V), calculated as the ratio of total migration distance to the experiment period. At least 3 independent experiments were performed for each condition and at least 50 cells were tracked for each experiment.

#### Cell morphology analysis

We calculated the cell’s aspect ratio using Fiji, an image processing package for ImageJ. Briefly, the cell boundary was segmented, and the best fitting ellipse of the cell boundary was calculated. The aspect ratio was calculated as the ratio of the major axis to the minor axis of the best fitting ellipse.

All parameters are presented as the average value ± standard error of the mean (SEM). Statistical analyses were performed with Student's *t*-test (for two samples) and ANOVA (for more than two samples). *p* < 0.05 (*) is considered significantly different.

## Results

### Development of a simple microfluidic chemotaxis device

In this study, a simple microfluidic device was developed for rapid and quantitative test of neutrophil chemotaxis (Fig 1A). The following features of this device make it suitable for chemotaxis testing in this study: 1) This device generates controlled and stable chemical gradient based on continuous laminar flow mixing in the main gradient channel. The gradient generation principle is similar to the previously characterized “Y” shape microfluidic gradient generator [24]; 2) This device does not require external pumps for fluid infusion. The flows are maintained by the pressure difference between the source inlet wells and the outlet well. Thus, this standalone device allows simple experiment setup, rapid gradient generation and chemotaxis testing; and 3) Similar to a previously reported design [35], this device uses a pressure balance zone to stabilize the flows from different source inlet wells. This design makes the downstream gradient generation insensitive to the variation in solution volume of the source inlet wells. Therefore, the requirement of assay operation accuracy is reduced. Comparing to the previous design that uses complicated downstream microfluidic channel networks, the current design is significantly simplified by using simple zigzag channels before and after the pressure balance zone. This design allows faster gradient generation.

We monitored the gradient stability in the main gradient channel by measuring the fluorescent intensity profile of FITC-dextran over 2 hrs. Our results show that a gradient was generated within the first minute after adding the solutions to the source inlet wells and was stable over the 2 hr period, which is sufficient for neutrophil chemotaxis experiment (Fig 1B). When a known chemoattractant, or the supernatant of a COPD patient sputum, was added to one of the inlet wells, it generated a chemoattractant gradient in the downstream gradient channel. Our results show clear neutrophil chemotaxis to the gradient (Fig 1C and 1D). The chemotaxis results will be described in the following sections.

### Characterizations of neutrophil chemotaxis to known chemoattractants using the microfluidic device

**Fig 2:**
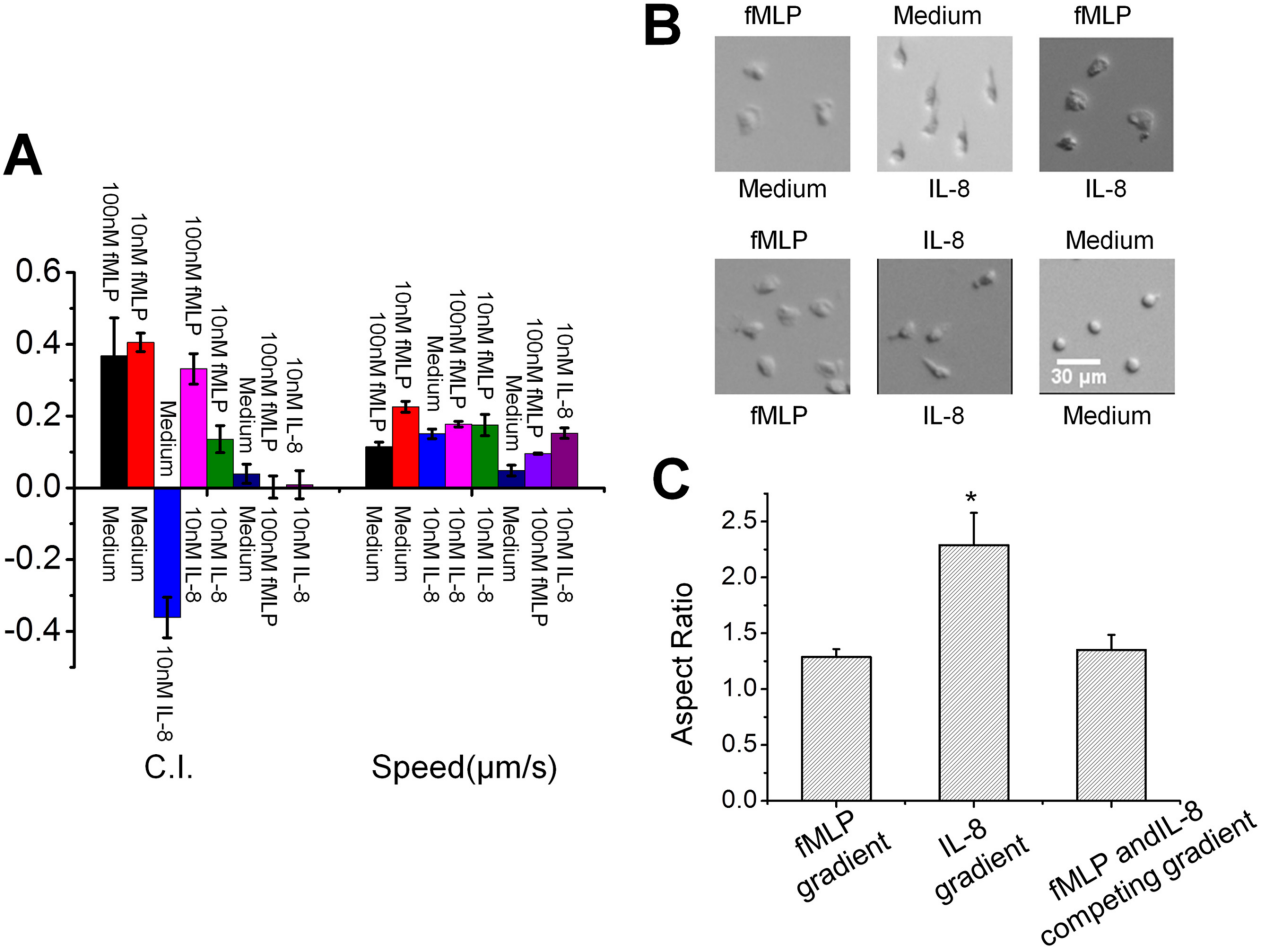
Neutrophil migration in different IL-8 and fMLP fields in the microfluidic device. **(A)** C.I. and migration speed in different IL-8 and fMLP fields; **(B)** Representative images of cell morphology in different IL-8 and fMLP fields; **(C)** Aspect ratio of cells in different IL-8 and fMLP gradients. Positive C.I. indicates cell migration towards the chemoattractant source labeled on the top; negative C.I. indicates cell migration towards the chemoattractant source labeled at the bottom.

We validated the developed microfluidic device by testing neutrophil chemotaxis to single and competing gradients of recombinant chemoattractants IL-8 (10 nM) and fMLP (10 nM and 100 nM). These selected IL-8 and fMLP concentrations were demonstrated to induce neutrophil chemotaxis and a clear dominating effect of fMLP over IL-8 in directing neutrophil migration in competing gradients of these two chemoatttractants [4, 31, 36]. In single IL-8 or fMLP gradients, neutrophils showed strong chemotaxis towards the gradient as measured by the Chemotactic Index (C.I.) (Fig 2A). The C.I. in 10 nM and 100 nM fMLP gradients was comparable. In competing gradients of IL-8 and fMLP that were configured in the opposite directions, neutrophils preferentially migrated towards fMLP (Fig 2A). This result is consistent with previous reports that demonstrated the dominant neturophil chemotaxis toward fMLP over IL-8 [36], and the underlying signaling hierarchy of fMLP over IL-8 in directing neutrophil chemotaxis [5]. However, the C.I. in competing gradients of 10 nM fMLP and 10 nM IL-8 was lower compared to it in competing gradients of 100 nM fMLP and 10 nM IL-8, suggesting the dose-dependent dominating effect of fMLP over IL-8. The C.I. is significantly lower in three control conditions (i.e. medium alone, uniform IL-8 field or uniform fMLP field)(Fig 2A). The migration speed was comparable in all gradient conditions and uniform 10 nM IL-8 field, but lower in medium control and 100 nM fMLP fields (Fig 2A).

In addition to directionality and motility measurements of neutrophil migration, we further compared the morphology of chemotaxing cells in different IL-8 and fMLP gradient conditions. In single IL-8 gradient, cells displayed a narrower cell front and elongated tail (Fig 2B). By contrast, in single fMLP gradients, cells displayed a wide cell front without clear formation of tail (Fig 2B). In both condition, the leading edge of the cells were towards the gradient direction. These observations are consistent with the previous reports in the literature [37]. Consistently, in competing gradients of IL-8 and fMLP, the cell morphology was similar to those displayed in single fMLP gradients (Fig 2B). In the medium control, cells showed round morphology without clear polarization (Fig 2B). In a uniform IL-8 or fMLP field, cells showed similar morphology to it in IL-8 or fMLP gradient, but with randomly polarized orientation (Fig 2B). The cell morphological difference was characterized by the aspect ratio measurement. Fig 2C shows the aspect ratio in different gradients. The aspect ratio of cells in uniform fMLP, uniform IL-8, and medium control were 1.19±0.06, 2.16±0.14, and 1.17±0.04 respectively.

Considering all the data, the microfluidic device was successfully validated with respect to measuring neutrophil chemotaxis in different gradient conditions with satisfying fidelity.

### Characterizations of neutrophil chemotaxis to sputum from COPD patients and healthy control subjects using the microfluidic device

**Fig 3:**
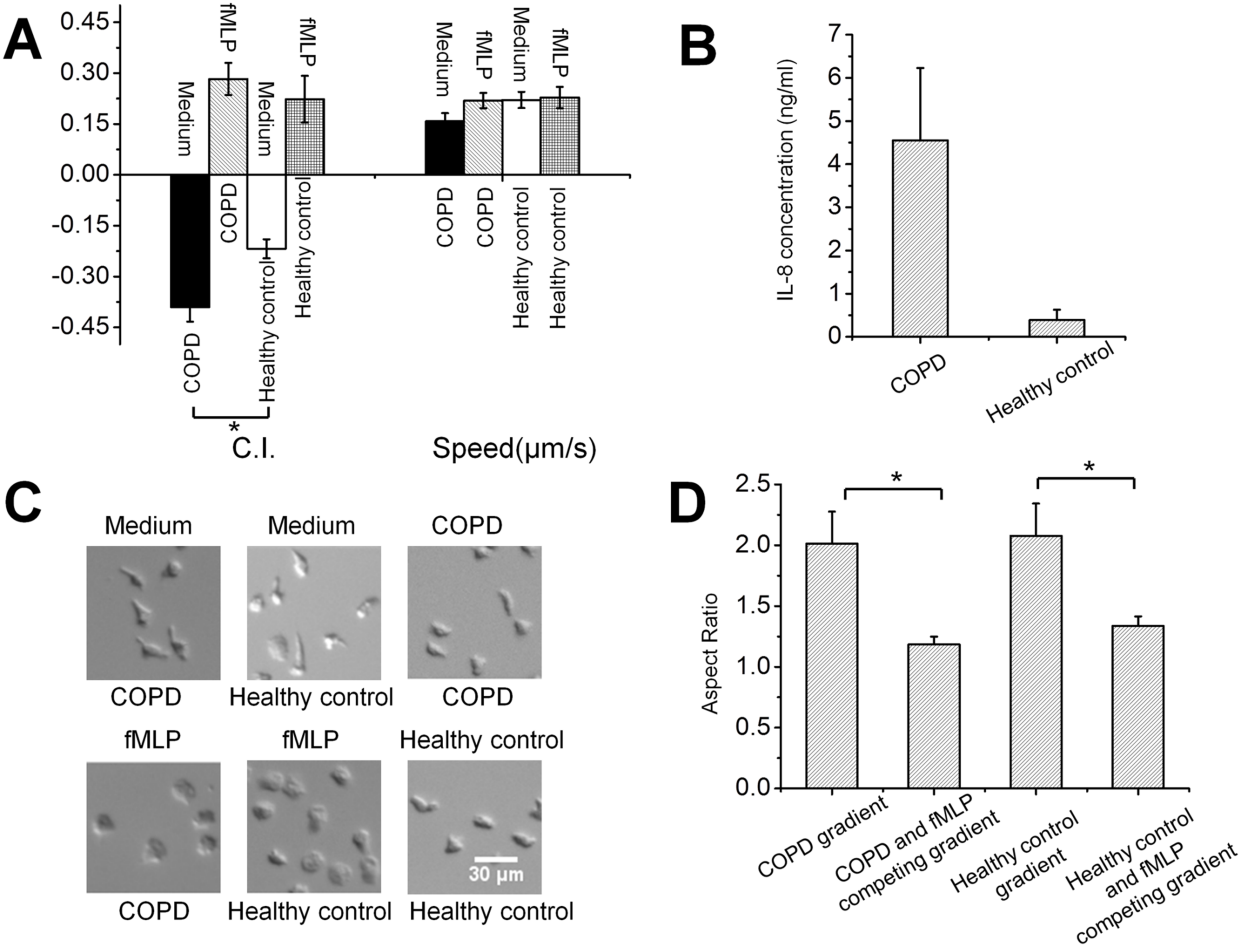
Neutrophil migration in different sputum (COPD or healthy control) and fMLP fields. **(A)** C.I. and migration speed in different sputum and fMLP fields; **(B)** IL-8 concentration in COPD sputum (n = 3) and healthy control sputum (n = 3); **(C)** Representative images of cell morphology in different sputum and fMLP fields. **(D)** Aspect ratio of cells in different sputum and fMLP gradients. Positive C.I. indicates cell migration towards the chemoattractant source labeled on the top; negative C.I. indicates cell migration towards the chemoattractant source labeled at the bottom.

We then used the microfluidic device to test neutrophil chemotaxis to sputum from COPD patients versus healthy control individuals. Our results showed that single COPD sputum gradients and single healthy control sputum gradients induced strong neutrophil chemotaxis as measured by the C.I. (Fig 3A). The levels of C.I. generated in response to the COPD sputum gradients were significantly higher compared with the levels of C.I. generated by the healthy control sputum gradients (Fig 3A). The migration speed is comparable (Fig 3A). These results suggest that neutrophil chemotaxis to a sputum gradient measured in our microfluidic device can distinguish COPD patients and healthy control subjects.

We further tested neutrophil chemotaxis in competing gradients of fMLP versus sputum from COPD patients or healthy control individuals. In competing gradients of fMLP and the COPD sputum, neutrophils preferentially migrated towards the fMLP gradient (Fig 3A). Similarly, in competing gradients of fMLP and the healthy control sputum, neutrophils also preferentially migrated towards the fMLP gradient (Fig 3A). The migration speeds were comparable (Fig 3A). Following the same analysis method as in the previous section, we compared the cell morphology in different sputum gradients. Our results revealed that the cell morphology in single COPD or healthy control sputum gradients was similar to the morphology of cells in IL-8 gradients (Fig 3C). In competing gradients of fMLP and sputum (COPD or healthy control), the cell morphology was similar to that of cells exposed to single fMLP gradients (Fig 3C). In a uniform COPD or healthy control sputum field, cells showed relatively less elongated morphology with random orientation compared to it in the single sputum gradient (Fig 3C). The cell morphology difference was quantitatively characterized by measuring the cell’s aspect ratio. Fig 3D shows the aspect ratio in different gradients. The aspect ratio of cells in uniform COPD sputum and uniform healthy control sputum were 1.58±0.19 and 1.46±0.05 respectively.

Collectively, these results suggest that neutrophil chemotaxis to sputum may be induced by different levels of tissue-derived chemoattractants such as IL-8 in the sputum. Consistently, ELISA measurements showed higher IL-8 level in the COPD sputum than it in the healthy control sputum (Fig 3B). This is in conceptual agreement with the previous report in the literature [11].

**Fig 4:**
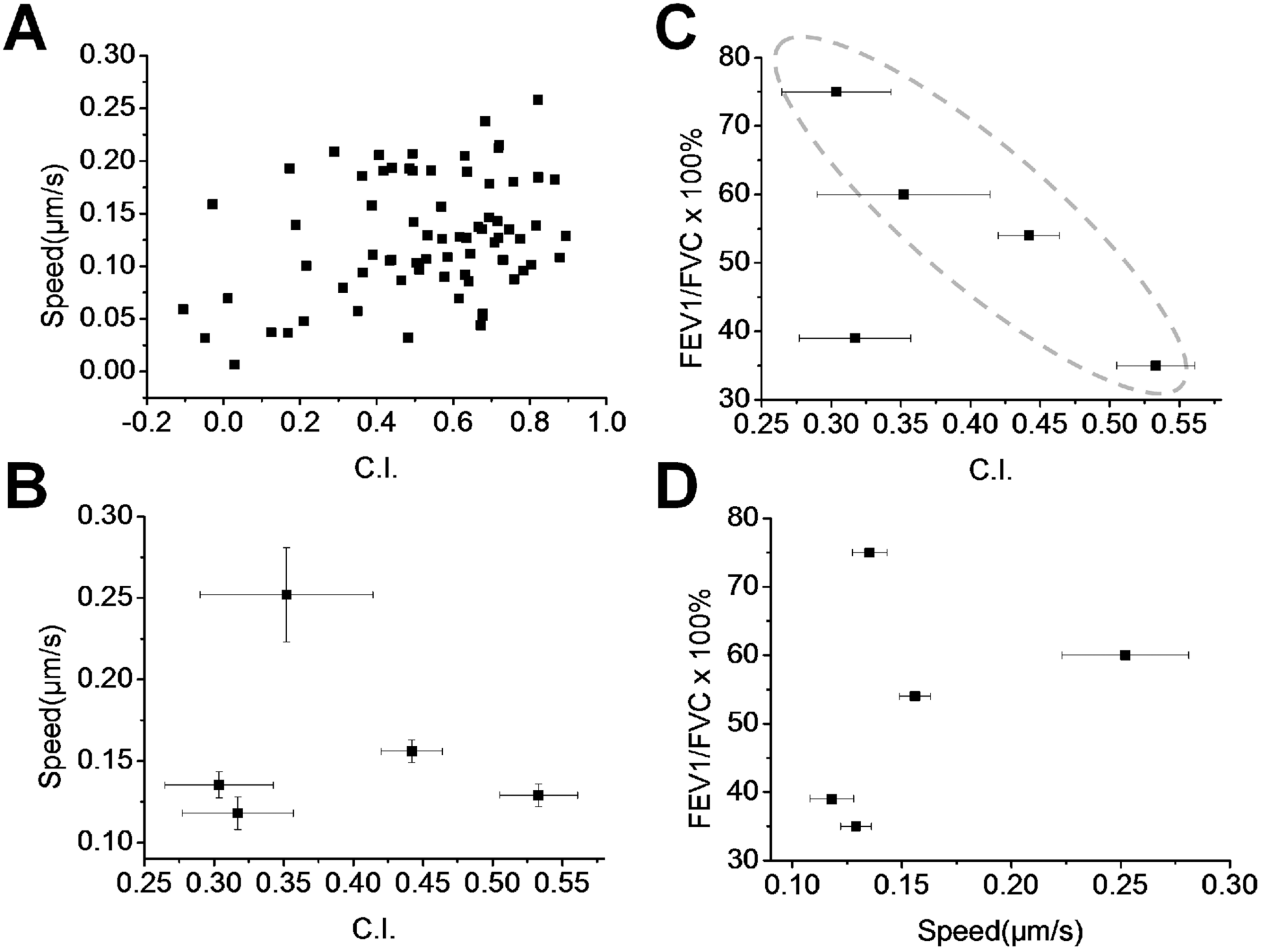
Correlations between neutrophil chemotaxis parameters and spirometry data. **(A)** No correlation is found between C.I. and migration speed in COPD sputum gradient from a single representative experiment; **(B)** No correlation is found between C.I. and migration speed from different COPD sputum samples; **(C)** Correlation between C.I. and FEV1/FVC. The dashed ellipse highlights the data with significant correlation; **(D)** No correlation is found between the migration speed and FEV1/FVC. The error bars indicate SEM.

Finally, we attempted to search for possible correlations between different neutrophil chemotaxis parameters (i.e. C.I. and the migration speed), and between the neutrophil chemotaxis parameters and the conventional COPD diagnosis parameter (i.e. the spirometry data). The clinical descriptors and chemotaxis measurements for the COPD patients in this study are summarized in Table 1. Our results show no correlation between C.I. and the speed for cells in the same experiment (Fig 4A) or for different COPD patients (Fig 4B and Table 1). Although the correlation between the C.I. and FEV1/FVC is weak using the data from all 5 patients, 4 out of the 5 COPD patients show clear correlation between the C.I. and FEV1/FVC (R^2^ = 0.93)(Fig 4C). Specifically, increased C.I. is correlated with decreased FEV1/FVC. The patient who did not follow this correlation has a younger age, a low FEV1/FVC as well as a low C.I. However, no correlation between the cell migration speed and FEV1/FVC is found (Fig 4D).

## Discussion

In this study, and for the first time, we established an effective microfluidic platform for quantitative analysis of neutrophil chemotaxis to COPD sputum samples. This is also the first time that clinical samples from chronic disease patients were used as chemoattractant source for microfluidics-based chemotaxis studies. Therefore, our results better reflect the migration and chemotaxis properties of neutrophils under pathological conditions.

Furthermore, our test results provide quantitative evidence, at the single cell level, for significantly different levels of neutrophil chemotaxis to sputum from COPD patients versus healthy control subjects. Our data also provide a range of quantitative characterizations of neutrophil migration and chemotaxis to COPD sputum, including directionality, motility, and cell migratory morphology. In addition to directly quantifying neutrophil chemotaxis to sputum, competition of sputum and defined chemoattractants in directing neutrophil migration provides another complementary measurement. These results offer a panel of quantitative descriptors based on neutrophil chemotaxis to discriminate COPD and non-COPD samples, and suggest the important chemotactic factors in the sputum sample responsible for neutrophil chemotaxis. On the other hand, it is worth noting that previously Yoshikawa *et al*. [21] showed impaired neutrophil chemotaxis to the COPD sputum compared with the healthy control sputum, which contradicts our results. This is possibly due to the difference between the traditional transwell assay used in the study by Yasikawa *et al* and the microfluidics-based method used in our study.

In contrast to an intriguing earlier study that studied neutrophil chemotaxis for asthma diagnosis using a microfluidic device [34], our results show an interesting negative correlation between the C.I. of neutrophils to the sputum versus spirometry data of COPD patients (4 out of 5 COPD patients). This result is consistent with the previously reported negative correlation between the IL-8 concentration and FEV1/FVC in COPD patients [11]. This suggests a possible correlation between neutrophil chemotaxis and the severity of COPD, and the potential of using the chemotaxis characteristics to identify COPD in a particular stage. Further testing with a larger cohort is required to clarify this correlation. In addition, it will be very interesting to investigate the potential diagnostic value of this new microfluidics-based test by correlating neutrophil chemotaxis to the patient’s medical history, such as the exacerbation record to improve disease group classification, risk prediction, and treatment strategy [38].

Microfluidic analysis of neutrophil chemotaxis has been recently demonstrated for successful diagnosis of asthma [34]. Our study provides another example of successful use of a microfluidic platform for diagnosis of inflammatory lung disease, by assessing neutrophil chemotaxis. These two studies suggest the promise of new biomarkers at the cellular function level for disease diagnosis and monitoring. On the other hand, microfluidic cell analysis often requires specialized research facilities, such as microfabrication and live cell microscopy labs, as well as highly-skilled personnel to perform the experiments and analyze the data. These requirements present significant challenges for adopting microfluidic methods for a routine test in clinical settings. To overcome these limitations, recent studies have demonstrated the promise of practical point-of-care applications based on microfluidic chemotaxis analysis [33]. In this direction, on-chip capture of neutrophils directly from a drop of blood, based on specific antibody or adhesion molecules for recognizing neutrophils, has been demonstrated [39, 40]. The captured cells can be directly used for chemotaxis analysis in the same microfluidic chip, which significantly reduces the current lengthy process for cell isolation and requires only minimal amounts of blood for each test. Pump-free standalone microfluidic gradient devices have been employed to minimize the requirement of external instrument controls, such as syringe pumps or other pressure sources. The microfluidic gradient generator used in this study provides highly controlled flow-based chemical gradients without external fluid perfusion instrument. It has the advantage in fast generation of stable gradients compared with free diffusion-based standalone microfluidic gradient devices [30, 41]. The relatively simple and compact design of this device has the scaling potential to enable high-throughput tests on a single chip by integrating multiple test units in parallel. In addition, various image analysis methods have been developed to allow automated single cell tracking analysis, thus eliminate the need of lengthy and laborious post-experiment tracking analysis and permit instant result report [33].

To ultimately enable point-of-care test, the entire microfluidic system including microfluidic device, gradient generation and calibration, on-chip cell capturing, environmental control, chemotaxis experiment, image acquisition and automated data analysis and result reporting should be integrated to meet the requirements in portability, cost factor, ease-of-operation and reliability for clinical use. Toward this direction, we have previously reported a compact microfluidic chemotaxis analysis system (i.e. UMCAS) with most of the above listed features and demonstrated its effective use for measuring neutrophil chemotaxis to an IL-8 gradient [42]. This UMCAS system was effectively used to test neutrophil chemotaxis to sputum samples and the results (reported in a separate article [43]) are consistent with the results in this study using traditional microscopy. Therefore, the microfluidic chemotaxis system has the potential for on-site clinical COPD diagnosis.

In the current study, we used neutrophils from a third-party healthy blood donor to compare their chemotaxis to different sputum samples from CODP or non-COPD patients. It has also been reported that neutrophil from COPD patients in different stage will have different chemotaxis behaviors [21]. Similar effect was also reported in other neutrophil chemotaxis mediated lung diseases such as asthma [44]. Therefore, chemotaxis of neutrophils from COPD or non-COPD patients to relevant chemoattractants such as IL-8 and LTB4 should be compared. Furthermore, it will be important to further compare chemotaxis of neutrophils from COPD or non-COPD patient to their own sputum samples, which will determine if there is any patient specific response. Previous studies using conventional cell migration assays have also showed that neutrophil chemotaxis to the sputum from COPD patient with Alpha-1 Antitrypsin Deficiency (A1AD) is higher comparing to the sputum from non-A1AD COPD patients [22]. The developed microfluidic method can be readily used to quantitatively assess the effect of A1AD under better controlled experimental conditions, which may enable discrimination of COPD patients with or without genetic disorders.

In conclusion, our study for the first time demonstrated the successful use of microfluidic system for assessing neutrophil chemotaxis to clinical samples from COPD patients, and suggested the potential of this new method to assist COPD diagnosis and monitoring at the point-of-care.
